# Epidemiology and Treatment Challenges of Acne, With Insights Into the Role of Dermocosmetics: An Expert Consensus From India—A Modified Delphi Method

**DOI:** 10.1111/jocd.71080

**Published:** 2026-07-22

**Authors:** Mukta Sachdev, Nina Madnani, Malavika Kohli, Koushik Lahiri, Anurag Tiwari, Kalpana Sarangi, Rajat Kandhari, Rajetha Damisetty, Ramesh Bhat, Delphine Kerob, Shefali Trasi‐Nerurkar

**Affiliations:** ^1^ M S Clinical Research Pvt Ltd Bangalore Karnataka India; ^2^ Manipal Hospital Bengaluru Karnataka India; ^3^ P.D. Hinduja National Hospital & MRC Mumbai Maharashtra India; ^4^ Breach Candy Hospital Mumbai Maharashtra India; ^5^ Jaslok Hospital Mumbai Maharashtra India; ^6^ Apollo Multi Speciality Hospitals Limited Kolkata West Bengal India; ^7^ Center for Skin Diseases and Laser Treatment Bhopal Madhya Pradesh India; ^8^ KMC Manipal Karnataka India; ^9^ Max Nanavati Hospital Mumbai Maharashtra India; ^10^ DKS Clinique Mumbai Maharashtra India; ^11^ Dr Kandhari's Skin and Dental Clinic New Delhi India; ^12^ Mohana Skin, Hair and Aesthetic Clinic Hyderabad India; ^13^ Father Muller Medical College Mangalore Karnataka India; ^14^ La Roche‐Posay Laboratoire Dermatologique Levallois‐Perret France; ^15^ La Piel Skin Clinic Mumbai Maharashtra India

**Keywords:** acne, dermocosmetics, expert consensus, hyperpigmentation, modified Delphi, treatment challenges

## Abstract

**Background:**

Acne vulgaris is a common skin condition that typically begins in adolescence but can often persist in adulthood, contributing to significant physical and psychosocial burden. Despite the availability of multiple treatment options, challenges such as poor adherence, treatment‐related side effects, and post‐inflammatory hyperpigmentation remain common in clinical practice. Dermocosmetics are increasingly used in acne management, either alone in mild cases or as adjuncts to medical therapy.

**Objectives:**

To assess acne prevalence, treatment practices, and key management challenges, along with the role of dermocosmetics, based on expert consensus from India.

**Methods:**

A panel of 10 dermatologists participated in a modified Delphi process comprising a pre‐meeting survey followed by a structured advisory board discussion, capturing clinical practice patterns, treatment approaches, and perspectives on dermocosmetic use in acne management.

**Results:**

Experts reported treating a high number of acne patients, primarily adolescents, with a higher proportion of females. Hyperpigmentation and scarring were common sequelae of acne, while treatment adherence and antibiotic resistance, particularly to erythromycin and azithromycin, were noted as key challenges. Procedural interventions, including chemical peels, lasers, and comedone extraction, were increasingly used in early acne management, especially in patients prone to post‐inflammatory hyperpigmentation and relapse. Low‐dose isotretinoin and hormonal therapies were commonly used in acne management, while dermocosmetics were utilized as monotherapy in mild cases and as maintenance therapy following treatment in more severe cases. Lifestyle factors such as high glycaemic diets, whey protein, multivitamins, and steroid‐like drugs were identified as potential contributors to acne exacerbation.

**Limitations:**

Findings are based on a small expert panel convened with industry support and reflect clinical opinion rather than primary patient data.

**Conclusion:**

Procedural interventions are becoming more popular, thereby providing an opportunity to incorporate dermocosmetics into acne treatment regimens.

## 1 Introduction

Acne vulgaris, a common inflammatory skin disease of the pilosebaceous unit [1, 2], affects approximately 9.4% of the global population [3]. In India, the overall prevalence of acne in children aged 11–19 years is 72.3% [4]. It is a chronic condition that may persist for several years, with a high rate of relapse, warranting prolonged treatment. Acne commonly affects adolescents aged 14–19 years [1], impacts approximately 85% of teenagers [5], and typically resolves by the mid‐twenties. It affects both genders, with a distinct subtype observed in women over the age of 25 years old and is more prevalent in males than females [6].

Acne vulgaris is a multifactorial condition arising from increased sebum production, abnormal keratinization of the pilosebaceous follicle, microbiome dysbiosis with an overabundance of *Cutibacterium acnes* (with certain phylotypes such as *C. acnes* IA1 being more virulent), and in situ inflammatory activity [7]. Clinically, it presents with primary lesions such as comedones, papules, and pustules, often accompanied by secondary lesions including scars, erythema, and hyperpigmentation. Acne lesions primarily occur on the face but may also appear on the chest, back, and shoulders, a presentation referred to as truncal acne. Truncal acne affects over half of acne patients but is often underreported and overlooked [8]. Although facial acne lesions are the primary reason for dermatological consultation, their psychological impact is frequently underestimated. Acne is associated with significant emotional and psychological burden, comparable to chronic conditions such as epilepsy, diabetes, and asthma, etc. often leading to negative self‐image and, in some cases, affecting career prospects [9, 10]. Research has shown that patients often feel “unheard” or “trivialized” by practitioners, resulting in dissatisfaction with care; therefore, a patient‐centered treatment approach is recommended [11].

Exposome factors such as diet, including consumption of dairy products, sweets, alcohol, whey protein, and high glycaemic load, as well as environmental factors such as hot and humid weather and sun exposure have been implicated in the development of acne vulgaris [8, 12, 13, 14]. In contrast, Omega‐3 fatty acids and γ‐linoleic acid have been associated with a reduction in acne prevalence [15].

Effective management of acne vulgaris involves an initial treatment phase aimed at reducing the severity and extent of lesions, followed by a maintenance phase to prevent relapses. Current acne management guidelines emphasize the use of combination therapy, with retinoid‐based topicals as first‐line treatment [16], either alone or in combination with antimicrobial agents [6].

The term dermocosmetics refers to a range of products that provide both active skincare and cosmetic benefits. Dermocosmetics are formulated with dermatologically active ingredients and evaluated through clinical studies, though they are not subject to the same regulatory approval process as pharmaceutical drugs [17]. Dermocosmetics target multiple aspects of acne pathophysiology and can be used as monotherapy in mild acne cases and as maintenance therapy after initial management. They can also act as adjuncts to pharmacologic and light/laser therapies, to minimize retinoid‐related side effects, enhance treatment efficacy, and support microbiome balance. Furthermore, they may contribute to improved patient satisfaction and adherence [18, 19]. Although they exert biological effects such as anti‐inflammatory, keratolytic, and antimicrobial actions, these effects are generally milder and slower compared to prescription medications.

An advisory board meeting was conducted in February 2025 with a panel of expert dermatologists to explore acne prevalence, treatment practices, and key management challenges, including treatment adherence, antibiotic resistance, and the role of dermocosmetics. This consensus is based on structured expert input and real‐world clinical insights and aims to provide an overview of the role of dermocosmetics as monotherapy, adjunctive, and maintenance strategies in acne management. It should be noted that dermocosmetics occupy a regulatory gray area globally and are not classified as pharmaceutical drugs. In India specifically, no intermediate regulatory category exists—products are regulated either as cosmetics or as drugs under the Drugs and Cosmetics Act, and dermocosmetics fall within the cosmetics category. They are therefore not subject to the same regulatory approval pathway as prescription medications, and efficacy claims are based on clinical studies rather than formal drug registration trials.

## 2 Materials and Methods

### 2.1 Panel Structure and Selection

This expert consensus was developed during an advisory board meeting on acne and the role of dermocosmetics, conducted in February 2025 in Jaipur, India, and organized by L'Oréal Dermatological Beauty. The panel comprised 10 experienced dermatologists from diverse geographic regions across India, including Bhopal, Delhi, Mumbai, Kolkata, Hyderabad, Mangalore, and Bangalore, ensuring representation of varied clinical practices and patient populations (Table 1).

**TABLE 1:** Panel demographics of participating dermatologists.

S. No.	Panelist	City	Years of experience
1.	Dr. Anurag Tiwari	Bhopal	> 20 years
2.	Dr. Kalpana Sarangi	Mumbai	> 20 years
3.	Dr. Koushik Lahiri	Kolkata	> 20 years
4.	Dr. Malavika Kohli	Mumbai	> 20 years
5.	Dr. Nina Madnani	Mumbai	> 20 years
6.	Dr. Rajat Kandhari	Delhi	> 20 years
7.	Dr. Rajetha Damisetty	Hyderabad	> 20 years
8.	Dr. Ramesh Bhat	Mangalore	> 20 years
9.	Dr. Shefali Trasi	Mumbai	10–20 years
10.	Dr. Mukta Sachdev (Moderator)	Bengaluru	> 20 years

*Note:* Experience bands are based on survey data (80% > 20 years, 20% 10–20 years). Individual years were not captured.

Panelists were selected based on their clinical expertise in acne management and regional representation. The meeting was facilitated by designated moderators, who guided the discussion, ensured structured participation, and supported the integration of survey findings with real‐world clinical perspectives. No patients or members of the public were involved in any stage of the study. The exercise was conducted exclusively among medical professionals to capture expert clinical insights.

### 2.2 Study Design and Consensus Methodology

**FIGURE 1:**
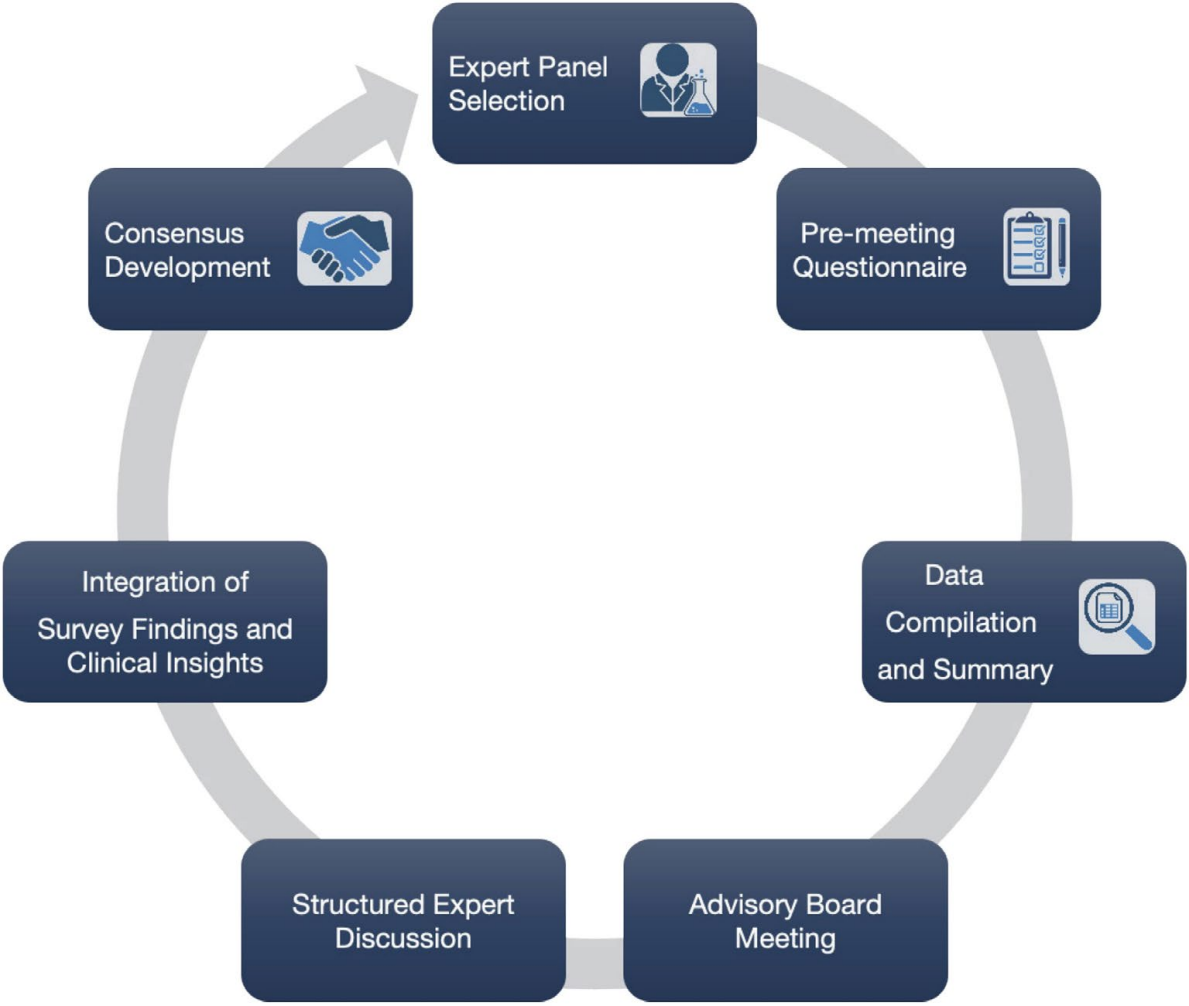
Schematic representation of the modified Delphi‐based consensus process used in this study.

A modified Delphi approach was employed, comprising three sequential stages (Figure 1).

In Stage 1 (pre‐meeting), panelists independently completed a structured 12‐item multiple‐choice questionnaire distributed by email prior to the meeting, without prior knowledge of other participants' responses, ensuring independence of initial input. Survey responses were aggregated and analyzed before the meeting.

In Stage 2 (Round 1 in‐meeting discussion), aggregated findings were presented to the panel and a structured moderator‐led discussion addressed five pre‐defined clinical questions such as (1) the frequency of facial versus truncal acne in clinical practice; (2) the most frequently prescribed drug treatments; (3) major challenges in acne management; (4) major challenges in patient adherence to treatment; and (5) the role of dermatological skincare in acne management.

In Stage 3, final consensus statements were derived through moderator‐facilitated agreement, with majority positions recorded and any persistent minority views documented.

The term “modified Delphi” reflects the adaptation of the traditional fully anonymous iterative process to incorporate a structured face‐to‐face discussion round in place of additional anonymous questionnaire iterations, a format previously validated in clinical expert consensus studies. Consensus was defined as agreement expressed by the majority of panelists during the moderated discussion phase.

### 2.3 Data Handling and Analysis

Data obtained from the pre‐meeting questionnaire were summarized using descriptive statistics. Qualitative insights from the advisory board discussion were thematically analyzed and integrated with survey findings to provide a comprehensive overview of clinical practice patterns and expert perspectives.

## 3 Results

The consensus meeting was conducted on February 7, 2025, in Jaipur, India, and comprised a pre‐meeting questionnaire followed by structured expert discussions. Key findings are presented under thematic subheadings, with summary data provided in Tables 2, 3, 4, and 5.

**TABLE 2:** Patient load and demographic characteristics of acne cases.

No.	Parameter	Findings
1.	Patients/week	10–50 (60%), 50–100 (20%), > 100 (20%)
2.	Age distribution	Mostly adolescents (60:40)
3.	Gender distribution	Female predominance (40%)

**TABLE 3:** Dermocosmetic usage patterns and commonly recommended ingredients.

S. No.	Parameter	Findings
1.	Frequent use	40%
2.	Sometimes	30%
3.	Always	30%
4.	Adjunct use	47%
5.	Common products	Cleansers, moisturizers, sunscreens
6.	Key ingredients	Salicylic acid, ceramides, niacinamide

**TABLE 4:** Distribution of commonly recommended dermocosmetic ingredients.

S. No.	Ingredient/Category	Responses (*n*)	% of total responses
1.	Skin barrier‐repairing agents (e.g., ceramides)	10	18%
2.	Moisturizing agents	9	16%
3.	Salicylic acid	6	11%
4.	Glycolic acid	6	11%
5.	Soothing agents	6	11%
6.	Other (niacinamide, sunscreen actives, etc.)	19	34%
	Total	56	100%

**TABLE 5:** Expert‐derived consensus insights on dermocosmetics in acne management.

No.	Key domain	Expert consensus insight
1.	Role in therapy	Dermocosmetics are widely used as adjuncts to pharmacological treatment and are also considered in maintenance therapy
2.	Clinical benefits	Dermocosmetics contribute to improved treatment adherence, better tolerability, and reduction of treatment‐related side effects
3.	Common product categories	Cleansers, moisturizers, and sunscreens are the most frequently recommended dermocosmetic products
4.	Key ingredients	Salicylic acid, ceramides, and niacinamide are commonly recommended based on expert practice
5.	Patient‐related factors	Treatment adherence is influenced by side effects, cost, and patient‐driven practices such as self‐medication
6.	Evolving practices	Increasing integration of dermocosmetics alongside procedural and pharmacological interventions in acne management

### 3.1 Acne Prevalence and Patient Demographics

Participating dermatologists reported managing a substantial number of acne patients in routine clinical practice. 60% of panelists reported seeing 10–50 acne patients per week, while 20% saw 50–100 and 20% saw more than 100 patients weekly.

Most experts reported a higher proportion of adolescent patients, with a commonly observed adolescent‐to‐adult ratio of approximately 60:40. The most common reported ratio of adolescent to adult acne patients was 60:40 (reported by 50% of panelists), though 30% reported seeing predominantly adult patients at a 30:70 ratio, reflecting the growing burden of adult acne in clinical practice. However, variability in patient distribution was noted, with some clinicians reporting higher adult acne prevalence.

A female predominance was reported by the majority of panelists, with female‐to‐male ratios ranging from 60:40 (40% of panelists) to 90:10 (10% of panelists). A summary of patient load and demographic characteristics is presented in Table 2.

Post‐inflammatory hyperpigmentation was reported as highly prevalent, with 70% of panelists estimating that 20%–50% of their acne patients presented with PIH, and a further 20% reporting this proportion exceeded 50%.

### 3.2 Treatment Adherence Challenges and Antibiotic Resistance

Treatment adherence was identified as a major challenge in acne management, with approximately 50% of patients reported to have poor adherence to prescribed regimens. Contributing factors included treatment‐related side effects such as dryness, irritation, and peeling associated with retinoids and isotretinoin, misconceptions regarding treatment duration, and financial constraints leading to incomplete adherence.

Self‐medication driven by social media and omission of supportive skincare products, including cleansers, moisturizers, and sunscreens, was also commonly reported.

Antibiotic resistance, particularly to erythromycin and azithromycin, was identified as an emerging concern, prompting a shift toward non‐antibiotic therapeutic approaches.

### 3.3 Evolving Treatment Practices in the Indian Scenario

Experts reported an increasing use of procedural interventions, including chemical peels, lasers, and comedone extraction, particularly in patients with relapsing acne and those prone to post‐inflammatory hyperpigmentation.

Hormonal therapies, including spironolactone, combined oral contraceptives, and metformin, were commonly utilized in selected patients following appropriate clinical evaluation.

### 3.4 Use of Dermocosmetics in Acne Management

Dermocosmetics were widely incorporated into acne management. Approximately 40% of panelists reported frequently prescribing dermocosmetics, while 30% prescribed them sometimes, and another 30% reported consistent use.

Commonly prescribed dermocosmetic categories included cleansers, moisturizers, and sunscreens. Combination use of these products was reported by 24% of panelists, while a smaller proportion recommended serums or specialized formulations such as barrier‐repair creams, exfoliants, anti‐pigmenting, and anti‐aging products.

Frequently recommended ingredients included salicylic acid–based cleansers and barrier‐repair formulations containing ceramides and niacinamide, along with sun protection products. Dermocosmetic usage patterns and commonly recommended ingredients are summarized in Table 3. When asked how dermocosmetics were used in acne management, 47% of panelists reported using them as adjuncts to topical and/or systemic drugs to complement their mode of action. Maintenance therapy after completion of drug treatment and adjunctive use to mitigate side effects were each reported by 21% of panelists, while monotherapy for mild acne was reported by 11%.

In terms of efficacy properties sought in dermocosmetics, soothing and calming properties (24%) and skin barrier‐rebalancing (24%) were the most commonly prioritized benefits, followed by anti‐inflammatory and antibacterial properties (22%), safety when used alongside drugs (16%), skin microbiome‐rebalancing (8%), sebum control (3%), and sunscreen or anti‐pigmentation properties (3%).

When asked about preferred dermocosmetic ingredients, panelists identified a broad range of ingredient categories from a total of 56 responses (multiple selections permitted). Skin barrier‐repairing agents were the most frequently selected category (18%; *n* = 10), followed by moisturizing agents (16%; *n* = 9), salicylic acid (11%; *n* = 6), glycolic acid (11%; *n* = 6), and soothing agents (11%; *n* = 6). Other ingredients constituted 34% of responses (*n* = 19), which included niacinamide and ceramides, as highlighted during the structured discussion (Table 4). These findings reflect a clinical preference for multi‐functional ingredients that address both acne pathophysiology and skin barrier support.

### 3.5 Patient Perceptions and Treatment Challenges

Experts reported that patients frequently rely on information from social media, leading to self‐medication and inappropriate product selection. A common observation was the discontinuation of prescribed therapies once visible improvement was achieved, contributing to incomplete treatment and increased risk of relapse.

The unsupervised use of products such as hydroquinone was also identified as a concern due to the risk of misuse and adverse effects. Unrealistic patient expectations further contributed to poor treatment adherence and suboptimal outcomes.

### 3.6 Expert Recommendations

Experts emphasized the importance of patient education, improved counseling, and increasing awareness regarding appropriate acne management. They highlighted the need to address a broader age spectrum of acne, including pre‐adolescent and adult populations, and to consider contributing factors such as lifestyle, hormonal influences, and metabolic conditions.

The role of dermocosmetics in routine clinical practice was reinforced, particularly as adjunctive and maintenance therapies, with a need for improved accessibility, affordability, and India‐specific clinical evidence. These findings were further supported by qualitative insights from expert discussions, which highlighted real‐world challenges such as poor treatment adherence, patient‐driven treatment modifications, and the increasing importance of dermocosmetics in improving tolerability and compliance in acne management. Key consensus‐based insights derived from both quantitative and qualitative inputs are summarized in Table 5.

## 4 Discussion

This consensus brought together experienced dermatologists from various regions across India, enabling the integration of diverse clinical perspectives relevant to the Indian population. The combination of a pre‐meeting questionnaire and structured expert discussions allowed the collection of quantitative data alongside qualitative insights into real‐world clinical practice patterns. The findings of this consensus highlight several key aspects of acne management in routine clinical practice. In addition to structured survey findings, expert discussions provided valuable qualitative insights into real‐world acne management in India. Dermatologists consistently highlighted challenges such as poor treatment adherence driven by side effects, cost constraints, and patient‐led treatment modifications influenced by social media. There was a strong consensus on the importance of dermocosmetics in improving treatment tolerability, supporting skin barrier function, and enhancing adherence, particularly in patients receiving retinoids or systemic therapies. Experts also emphasized the increasing role of procedural interventions and the need for integrated treatment approaches combining pharmacological, procedural, and supportive skincare strategies.

Dermocosmetics are increasingly incorporated into acne management, both as monotherapy in mild cases and as adjuncts to pharmacologic treatments. As monotherapy, dermocosmetics containing multi‐targeting ingredients such as keratolytics, anti‐inflammatory agents, sebum‐regulating agents, and microbiome‐modulating components may be considered for mild acne. Previous studies have demonstrated their effectiveness in improving acne outcomes and reducing bacterial counts [20, 21, 22], while also supporting maintenance of skin microbiome diversity in mild acne [23]. These products have been reported to be well tolerated across different skin phototypes [24] and may contribute to reductions in acne lesions, improvements in global assessment scores [18, 19, 25], and reduction of post‐acne hyperpigmentation [26]. Following discontinuation of pharmacological treatment in moderate‐to‐severe acne, dermocosmetics may also support maintenance of treatment response [27, 28].

Dermocosmetics can also be used as adjuncts to pharmacological agents such as benzoyl peroxide [21, 28], and adapalene [29], where they may complement therapeutic effects, improve tolerability, and enhance patient satisfaction. Some dermocosmetic formulations have demonstrated comparable reductions in acne lesion counts to benzoyl peroxide 5% in controlled studies; however, independent replication of these findings is needed before definitive equivalence claims can be made.

**FIGURE 2:**
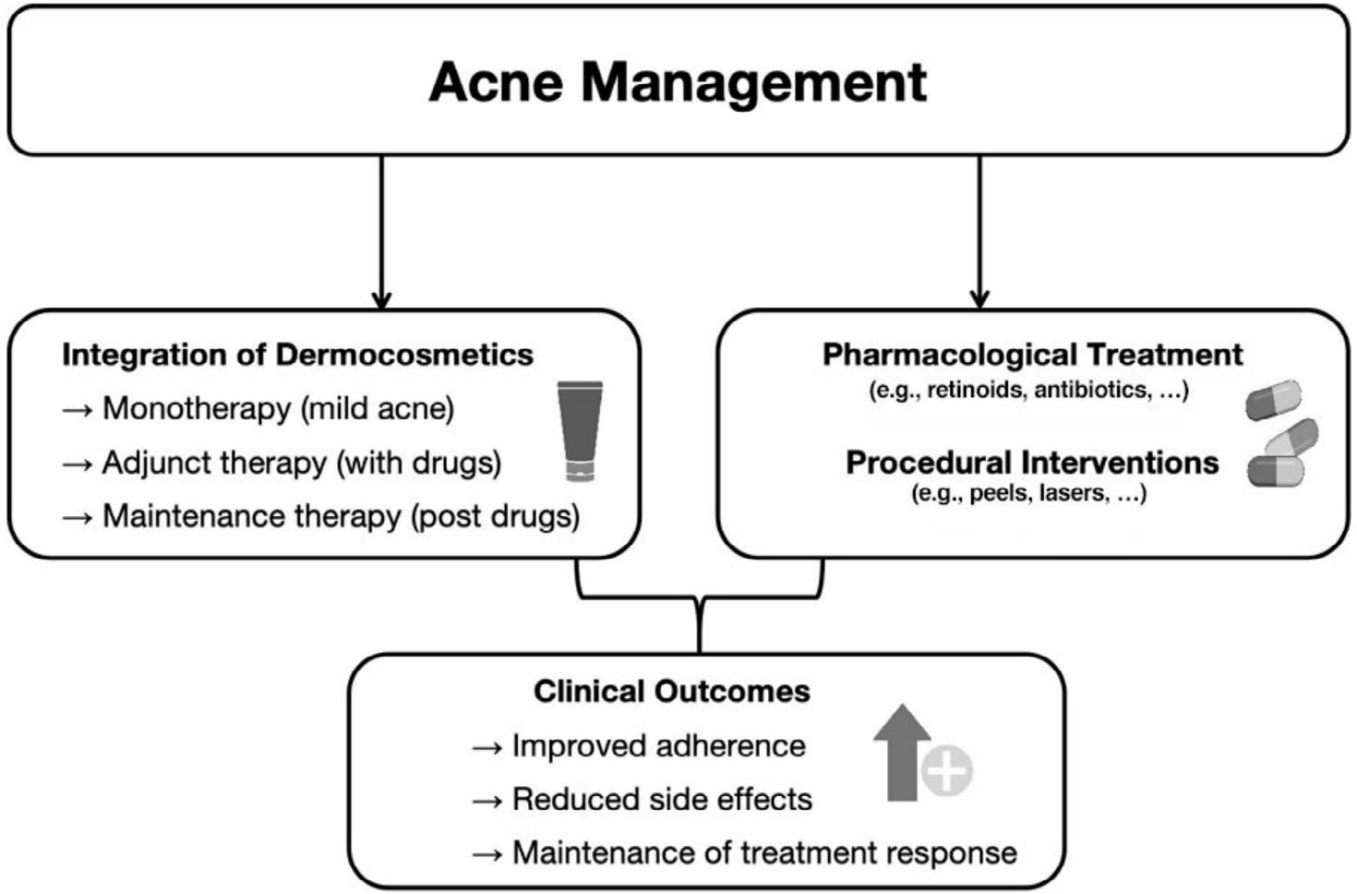
Conceptual overview of the role of dermocosmetics in acne management as monotherapy, adjunctive, and maintenance strategies.

As adjunctive therapy, dermocosmetics may help improve adherence to treatment, support skin barrier function [18, 30], and mitigate treatment‐related side effects [19, 31, 32, 33, 34]. One of the key observations from this consensus was that acne remains a common reason for dermatology consultations in India, with most clinicians managing a high patient volume. Additionally, procedural interventions are increasingly used in early acne management, creating opportunities to integrate dermocosmetics into pre‐ and post‐procedural care. The integrated role of dermocosmetics in acne management as monotherapy, adjunctive, and maintenance strategies is illustrated in Figure 2.

While dermocosmetics offer meaningful supportive benefits in acne management, their role must be appropriately contextualized. In moderate‐to‐severe acne, dermocosmetics alone are insufficient and should not replace pharmacological treatment. Their biological effects are generally milder and slower in onset compared to prescription medications, and the majority of available evidence derives from studies of limited sample size or those conducted within the context of specific product development programs. Furthermore, as highlighted during expert discussions, a key clinical challenge is the tendency of patients to discontinue prescribed treatments upon visible improvement and rely solely on dermocosmetics—a practice that increases the risk of relapse. Clinicians should therefore clearly communicate that dermocosmetics serve as adjuncts or maintenance tools within a broader, physician‐directed treatment strategy.

The discussion also emphasized the need for more India‐specific evidence on dermocosmetics, considering the diversity in skin types, climatic conditions, and environmental factors such as pollution.

## 5 Conclusion

Procedural interventions are increasingly utilized in acne management, reflecting evolving clinical practices. This trend provides an opportunity to incorporate dermocosmetics as supportive components within treatment regimens, particularly as adjunctive and maintenance therapies.

## 6 Limitations

This consensus statement has several limitations that should be considered when interpreting the findings. First, the panel comprised 10 dermatologists which, while geographically diverse, represent a small sample and may not reflect the full breadth of clinical practice across India's diverse patient populations and healthcare settings. Second, the advisory board meeting was organized and funded by L'Oréal Dermatological Beauty, a company with commercial interests in dermocosmetic products; despite the independence of clinical consensus discussions from the product presentation component, this sponsorship represents a potential source of bias that readers should consider. Third, the modified Delphi methodology employed here—comprising a pre‐meeting survey and a single structured in‐meeting discussion round—differs from traditional multi‐round Delphi processes with formal iterative anonymous measurement, which limits the rigor of consensus determination. Fourth, findings are based on expert opinion and self‐reported clinical practice patterns rather than primary patient data, and may not be generalizable to all populations, practice settings, or geographic regions outside India. Finally, the absence of formal quantitative consensus thresholds means agreement levels for individual statements cannot be precisely reported.

## Author Contributions

**Mukta Sachdev:** critical review for intellectual content. Reviewing manuscript content for scientific merit and coherence. Interpretation of existing literature, formulation of consensus statements, and review of the manuscript. **Nina Madnani and Delphine Kerob:** critical review for intellectual content. Reviewing manuscript content for scientific merit and coherence. **Malavika Kohli, Koushik Lahiri, Anurag Tiwari**, **Kalpana Sarangi**, **Rajat Kandhari**, **Rajetha Damisetty**, **Ramesh Bhat, Shefali Trasi‐Nerurkar:** reviewing manuscript content for scientific merit and coherence.

## Funding

The advisory board meeting was funded and organized by L'Oréal Dermatological Beauty. The sponsor had no role in the design of the pre‐meeting survey, analysis of survey data, moderation of clinical discussions, or preparation of this manuscript. All consensus statements reflect the independent clinical opinions of the expert panelists and are based solely on the clinical discussion component of the meeting.

## Ethics Statement

The authors have nothing to report.

## Conflicts of Interest

All authors received honoraria from L'Oréal Dermatological Beauty for participation in the advisory board meeting. The meeting included a product presentation component by the sponsor; however, this component did not form part of the consensus methodology reported here. Authors declare no other conflicts of interest relevant to this publication.

## Data Availability

The data supporting the findings of this study are available from the corresponding author upon reasonable request.
